# Dipole Theory of Polyzwitterion Microgels and Gels

**DOI:** 10.3390/gels10060393

**Published:** 2024-06-11

**Authors:** Murugappan Muthukumar

**Affiliations:** Department of Polymer Science and Engineering, University of Massachusetts, Amherst, MA 01003, USA; muthu@umass.edu

**Keywords:** polyzwitterions, hydrogels, gel swelling equilibria, hierarchical gel dynamics, dipole-dipole interactions, mesomorphic state, polyzwitterion gels

## Abstract

The behavior of polyzwitterions, constituted by dipole-like zwitterionic monomers, is significantly different from that of uniformly charged polyelectrolytes. The origin of this difference lies in the intrinsic capacity of polyzwitterions to self-associate intramolecularly and associate with interpenetrating chains driven by dominant dipolar interactions. Earlier attempts to treat polyzwitterions implicitly assume that the dipoles of zwitterion monomers are randomly oriented. At ambient temperatures, the dipolar zwitterion monomers can readily align with each other generating quadrupoles and other multipoles and thus generating heterogeneous structures even in homogeneous solutions. Towards an attempt to understand the role of such dipolar associations, we present a mean field theory of solutions of polyzwitterions. Generally, we delineate a high-temperature regime where the zwitterion dipoles are randomly oriented from a low-temperature regime where quadrupole formation is significantly prevalent. We present closed-form formulas for: (1) Coil-globule transition in the low-temperature regime, the anti-polyelectrolyte effect of chain expansion upon addition of low molar mass salt, and chain relaxation times in dilute solutions. (2) Spontaneous formation of a mesomorphic state at the borderline between the high-temperature and low-temperature regimes and its characteristics. A universal law is presented for the radius of gyration of the microgel, as a proportionality to one-sixth power of the polymer concentration. (3) Swelling equilibrium of chemically cross-linked polyzwitterion gels in both the high temperature and low-temperature regimes. Addressing the hierarchical internal dynamics of polyzwitterion gels, we present a general stretched exponential law for the time-correlation function of gel displacement vector, that can be measured in dynamic light scattering experiments. The present theory is of direct experimental relevance and additional theoretical developments to all polyzwitterion systems, and generally to biological macromolecular systems such as intrinsically disordered proteins.

## 1. Introduction

Polyzwitterions, where every repeat unit along the chain backbone is a zwitterion (electric dipole) consisting of positive and negative charges separated by designable distance *ℓ*, exhibit a wide range of material properties in the areas of biolubricants, cosmetics, soft contact lens, etc. [1,2,3,4,5,6,7,8,9,10]. Polyzwitterions are known to possess terrific material properties in the areas of cosmetics, soft contact lens, hemodialysis membranes, biolubricants, etc. The self-assembled structures by polyzwitterions hold potential applications. Gels from polyzwitterions are of tremendous use in therapeutic situations involving higher salinity (eye, for example), due to salt-philicity and the enhanced water-holding ability. Control of gel swelling by chemistry in these amphiphilic biocompatible materials can have significant impact. Self-assembly from polyzwitterions can also be of great use in encapsulating a variety of analytes such as drugs and fragrances. Yet, fundamental understanding of their structural organization and functions, such as their enhanced hydro- and salt-philicity in combination with amphiphilicity, is lacking.

In general, the polyzwitterion behavior depends on the dipole density along the chains, dipole length, and identities of the charge of the dipole [11,12,13,14,15,16,17,18,19,20,21,22,23,24,25,26,27,28,29,30,31,32], and has attracted significant theoretical and modeling efforts [33,34,35,36,37,38,39,40,41,42,43]. The rich functional properties of polyzwitterions (polydipoles), that are unprecedented in polyelectrolytes (polymonopoles), originate from delicate cooperative organization of dipolar local charge separations embedded along polymer backbone. The electrostatic forces from dipoles are gentler than the stronger Coulomb forces among monopole charges, resulting in fragility and diversity of local aggregation of zwitterions. The occurrence of dipolar interactions is ubiquitous in many biological macromolecular systems. Almost all protein molecules contain amino acid monomers that can possess either permanent or induced dipole moment. Furthermore, even for the nominal uniformly charged polyelectrolytes such as DNA and sodium poly(styrene sulfonate), dipolar forces are prevalent due to the unavoidable ion-pair formation arising from counterion adsorption on the repeat units. In general, dipole-dipole, charge-charge, and dipole-charge interactions are scripted in these biological as well as synthetic macromolecules resulting in rich complex structural and dynamical behavior. While there has been extensive attention to charge-charge interactions in treating charged macromolecules, the role of dipolar interactions among different parts of the polymer chains and their correlations accompanying physical aggregation is yet be conceptually framed and understood. In this general premise, it is desirable to consider the asymptotic limit of charged macromolecules, where only dipole-dipole interactions are present, as in the case of polyzwitterions. It is thus of fundamental interest to formulate a theoretical platform to account for the role of dipolar forces in polyzwitterions, which is the primary goal of this paper.

**Figure 1:**
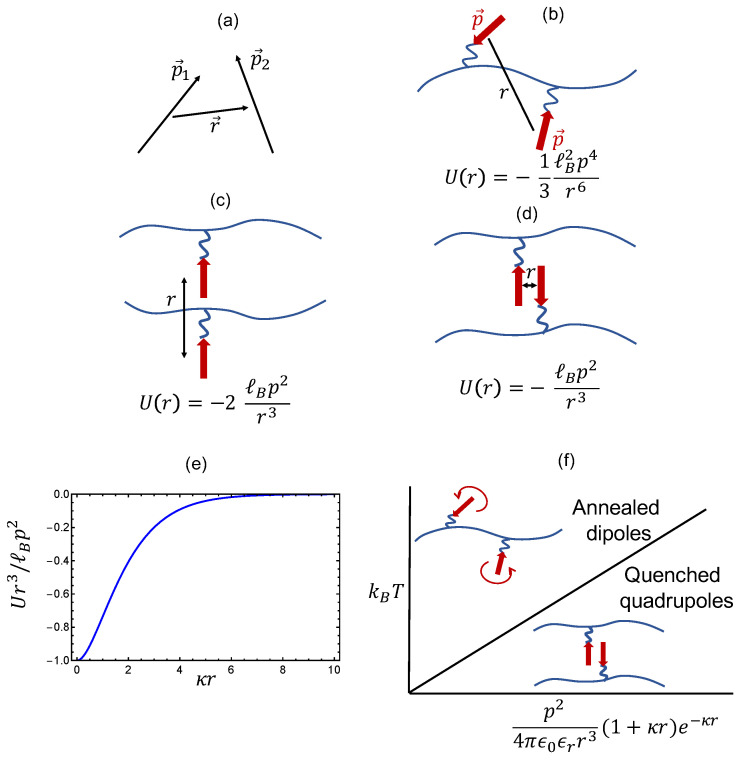
Different scenarios of dipole-dipole interactions as described in the main text. (**a**) Two dipoles $p_{1}$ and $p_{2}$ separated by a distance r. (**b**) Two dipoles of equal dipole moment *p* are randomly oriented and the interaction energy, $U\left(r\right)∼-1/r^{6}$ as noted. $\ell_{B}$ is the Bjerrum length. (**c**) Parallel orientation of two dipoles with interaction energy $U\left(r\right)∼-1/r^{3}$. (**d**) Anti-parallel orientation of two dipoles with $U\left(r\right)∼-1/r^{3}$. (**e**) Weakening of quenched dipole-dipole interaction with an increase in added salt concentration $c_{s}$, where the inverse Debye length $\kappa ∼\sqrt{c_{s}}$. (**f**) Demarkation of the high-temperature and low-tenperature regimes depending on p,r,κ, and the dielectric constant $\epsilon_{r}$ ($\epsilon_{0}$ = vacuum permittivity).

Towards a general treatment of effects emanating from zwitterions embedded on polymer backbones, let us first consider different configurations of dipoles (zwitterions) as illustrated in Figure 1. To begin with, the free energy of interaction of two dipoles of moments $p_{1}$ and $p_{2}$ separated by a distance r (Figure 1a) in a uniform dielectric medium is given by [1]

(1) $$\Delta F=\frac{1}{4\pi \epsilon_{0}\epsilon_{r}}\frac{1}{r^{3}}\left[p_{1}\cdot p_{2}-3\left(\hat{r}\cdot p_{1}\right)\left(\hat{r}\cdot p_{2}\right)\right],$$

where $\epsilon_{0}$ is the permittivity of vacuum, $\epsilon_{r}$ is the temperature-dependent dielectric constant of the medium, and the magnitude and unit vector of r are denoted by *r* and $\hat{r}$, respectively. Thus, the interaction between a pair of dipoles depends on their orientations and separation distance. This dipole-dipole interaction is screened in an electrolyte solution. Assuming the validity of the Debye-Hückel theory for electrolyte solutions, the above expression for ΔF is modified to [1]

(2) $$\Delta F=\frac{1}{4\pi \epsilon_{0}\epsilon }\frac{e^{-\kappa r}}{r^{3}}\left[\left(1+\kappa r\right)p_{1}\cdot p_{2}-\left(3+3\kappa r+\kappa^{2}r^{2}\right)\left(\hat{r}\cdot p_{1}\right)\left(\hat{r}\cdot p_{2}\right)\right],$$

where κ is the inverse Debye length (proportional to the square root of concentration $c_{s}$ of added salt in the solution). For monovalent salts,

(3) $$\kappa^{2}=4\pi \ell_{B}\left(c_{+}+c_{-}\right)=\left(8000\pi \ell_{B}N_{A}\right)c_{s},$$

where $c_{+}$ and $c_{-}$ are the number concentrations of dissociated cations and anions, $N_{A}$ is the Avogadro number, and $\ell_{B}$ is the Bjerrum length defined by

(4) $$\ell_{B}=\frac{e^{2}}{4\pi \epsilon_{0}\epsilon_{r}k_{B}T},$$

with $k_{B}T$ as the Boltzmann constant times absolute temperature. In the present theory, effects [1] from the finite size of dissociated ions are not accounted for.

If the polyzwitterion solution is dilute and the chain backbone is highly flexible, and if the temperature is high enough in comparison with local dipole-dipole interaction energy, the dipoles of the repeat monomers are expected to be randomly oriented (Figure 1b). The interaction free energy $u_{dd,\mathrm{random}}\left(r\right)$ (in units of $k_{B}T$) between two randomly oriented dipoles with the same dipole moment pe=ℓe (*p* is dipole length and *e* is the electronic charge), separated by distance *r* in a salty aqueous solution follows from Equation (2) after averaging over orientations of the dipoles as [1]

(5) $$U_{dd,\mathrm{random}}\left(r\right)=-\frac{1}{3}\frac{\ell_{B}^{2}p^{4}}{r^{6}}e^{-2\kappa r}\left[1+2\kappa r+\frac{5}{3}{\left(\kappa r\right)}^{2}+\frac{2}{3}{\left(\kappa r\right)}^{3}+\frac{1}{6}{\left(\kappa r\right)}^{4}\right].$$

If the temperature is not too high and if the salt concentration is not high enough to significantly screen the dipole-dipole interactions, then local pairing of dipoles will result in quadrupoles. There are primarily two orientations of the dipoles forming quadrupoles that are stable, namely the parallel and antiparallel configurations as shown in Figure 1c and Figure 1d, respectively. The distance vector r between the centers of the dipoles is along the same direction of the dipole orientations in Figure 1c, whereas it is orthogonal to the directions of the dipoles in Figure 1d. Using Equation (2), the pairwise dipole-dipole interaction energy (in units of $k_{B}T$) for these two types of quadrupoles is given by

(6) $$U_{dd,\mathrm{parallel}}\left(r\right)=-\frac{\ell_{B}p^{2}}{r^{3}}e^{-\kappa r}\left(2+2\kappa r+\kappa^{2}r^{2}\right),$$

and

(7) $$U_{dd,\mathrm{antiparsallel}}\left(r\right)=-\frac{\ell_{B}p^{2}}{r^{3}}e^{-\kappa r}\left(1+\kappa r\right).$$

Even though both parallel and antiprallel dipole orientations are stable, the parallel configuration of dipoles from chain backbones is expected to be considerably less stable (compared to the anti-parallel configuration) due to steric hindrance arising from chain backbone and the inter-dipole distance being much larger and hence weaker because of $1/r^{3}$ dependence (as sketched in Figure 1c,d). Therefore, it is reasonable to focus only on the anti-parallel arrangement of dipoles for the formation of quadrupoles in polyzwitterions. The strength of $u_{dd,\mathrm{antiprallel}}$ decreases with an increase $c_{s}$ as illustrated in Figure 1e, where $u_{dd,\mathrm{antiprallel}}$ is plotted against the inverse Debye length $\kappa ∼\sqrt{c_{s}}$.

At any salt concentration, there exists a threshold temperature $T_{\mathrm{threshold}}$ below which these antiparallel quadrupoles would spontaneously form as essentially quenched configurations, and above which random orientations of dipoles might become plausible. The threshold temperature is obtained from Equations (4) and (7) as

(8) $$T_{\mathrm{threshold}}=\frac{e^{2}p^{2}}{4\pi \epsilon_{0}\epsilon_{r}k_{B}r^{3}}e^{-\kappa r}\left(1+\kappa r\right).$$

As illustrated in Figure 1f, for temperatures higher than $T_{\mathrm{threshold}}$, the polyzwitterion chains behave as an annealed system with random orientation of zwitterions whereas they will form quenched and physically associated quadrupoles for temperatures below $T_{\mathrm{threshold}}$. The value of $T_{\mathrm{threshold}}$ depends on the magnitude of the dipole moment of the zwitterion, dielectric constant, and the salt concentration. Previous theoretical attempts to treat polyzwitterions, with the exception of the author’s previous work [35], deal with the high-temperature situation of random orientations of the dipoles. In the present paper, we will address the role of quenched configurations corresponding to the lower part of the diagram in Figure 1f, in addressing polyzwitterion gels. As discussed below, for temperatures above but close to $T_{\mathrm{threshold}}$, the quenched quadrupoles can result in an intermediate mesomorphic microgel formation where the quadrupoles function as physical cross-links and the intervening strands undergo conformational fluctuations with their dipoles being randomly oriented.

The rest of the paper is organized as follows. Defining a continuous Kuhn chain model encompassing chain connectivity and inter-segment dipole-dipole interactions in Section 2, the size and dynamics of isolated polyzwitterion chains are presented in Section 3. Self-assembly of mesomorphic polyzwitterion microgels at $T\geq T_{\mathrm{threshold}}$ is described in Section 4. Swelling equilibrium and hierarchical internal dynamics of polyzwitterion gels are described in Section 5, followed by concluding remarks on the phase behavior of polyzwitterion systems.

## 2. Model

Consider a system of *n* polyzwitterion chains each containing *N* repeat units, $n_{\gamma }$ ions of species γ from dissolved salt, and $n_{s}$ solvent molecules in volume *V*. Let *ℓ* and p be the linear size and dipole moment, respectively, of each zwitterionic repeat unit. Let also the dipole length of the zwitterion be *p* so that the magnitude of the dipole moment is |p|=pe, where the unit charge is explicitly expressed. Representing the polymer chains as continuous curves of contour length L=Nℓ, the Helmholtz free energy *F* of the system is given by

$$e^{-\frac{F}{k_{B}T}}=\frac{1}{n!n_{s}!\prod_{\gamma }n_{\gamma }!}\int \prod_{\alpha =1}^{n}D\left[R_{\alpha }\right]\int \prod_{i}^{n_{s}+\sum_{\gamma }n_{\gamma }}dr_{i}$$

$$\times \exp\{-\frac{3}{2\ell^{2}}\sum_{\alpha =1}^{n}\int_{0}^{N}ds_{\alpha }{\left(\frac{\partial R_{\alpha }\left(s_{\alpha }\right)}{\partial s_{\alpha }}\right)}^{2}-\frac{1}{2}\sum_{\alpha =1}^{n}\sum_{\beta =1}^{n}\int_{0}^{N}ds_{\alpha }\int_{0}^{N}ds_{\beta }U_{pp}\left[R_{\alpha }\left(s_{\alpha }\right)-R_{\beta }\left(s_{\beta }\right)\right]$$

$$-\sum_{\alpha =1}^{n}\int_{0}^{N}ds_{\alpha }\sum_{i=1}^{n_{s}}U_{ps}\left[R_{\alpha }\left(s_{\alpha }\right)-r_{i}\right]-\frac{1}{2}\sum_{i=1}^{n_{s}}\sum_{j=1}^{n_{s}}U_{ss}\left(r_{i}-r_{j}\right)$$

(9) $$-\sum_{\alpha =1}^{n}\int_{0}^{N}ds_{\alpha }\sum_{i=1}^{\sum_{\gamma }n_{\gamma }}U_{pi}\left[R_{\alpha }\left(s_{\alpha }\right)-r_{i}\right]-\frac{1}{2}\sum_{i=1}^{\sum_{\gamma }n_{\gamma }}\sum_{j=1}^{\sum_{\gamma }n_{\gamma }}U_{ij}\left(r_{i}-r_{j}\right)\}.$$

Here $R_{\alpha }\left(s_{\alpha }\right)$ is the position vector of the arc length variable $s_{\alpha }\left(0\leq s_{\alpha }\leq N\right)$ of the α-th chain. $U_{pp}\left(r\right)$ is the interaction energy between two repeat units of the chains separated by a distance r,

(10) $$U_{pp}\left(r\right)=v_{pp}\ell^{3}\delta \left(r\right)+v_{dipole}\left(r\right),$$

where $v_{pp}$ is the short-ranged monomer-monomer excluded volume interaction parameter, and δ(r) is the Dirac delta function. $v_{pp}$ represents only two-body interactions, and three-body interactions need to be included in describing situations where polyzwitterion chains form globule-like structures. $v_{dipole}\left(r\right)$ is the dipole-dipole interaction that depends on specific dipole orientations as displayed in Figure 1.

The short-ranged interactions between polymer repeat units and solvent molecules and between solvent molecules are given by

(11) $$U_{ps}\left(r\right)=v_{ps}\ell^{3}\delta \left(r\right)\;\;\;\;\;\;\;\;\;\;\mathrm{and}\;\;\;\;\;\;\;\;\;\;U_{ss}\left(r\right)=v_{ss}\ell^{3}\delta \left(r\right),$$

where $v_{ps}$ and $v_{ss}$ are parameters analogous to $v_{pp}$. The electrostatic interactions between dissociated monovalent ions and between dipoles and ions are given by

(12) $$U_{ij}\left(r\right)=\frac{z_{i}z_{j}\ell_{B}}{r}\;\;\;\;\;\;\;\;\;\;\mathrm{and}\;\;\;\;\;\;\;\;\;\;U_{pi}\left(r\right)=\frac{z_{i}}{e}\frac{\ell_{B}}{r^{3}}\left(p\cdot r\right),$$

where $z_{i}e$ is the charge of the *i*-th dissociated ion, p is the dipole moment of the repeat unit, and r=|r|.

Integrating out all degrees of freedom associated with mobile salt ions and solvent molecules within the framework of the Debye-Hückel theory, we get

$$e^{-\frac{F}{k_{B}T}}=e^{-\frac{F_{0}}{k_{B}T}}\frac{1}{n!}\int \prod_{\alpha =1}^{n}D\left[R_{\alpha }\right]\exp\{-\frac{3}{2\ell^{2}}\sum_{\alpha =1}^{n}\int_{0}^{N}ds_{\alpha }{\left(\frac{\partial R_{\alpha }\left(s_{\alpha }\right)}{\partial s_{\alpha }}\right)}^{2}$$

(13) $$-\frac{1}{2}\sum_{\alpha =1}^{n}\sum_{\beta =1}^{n}\int_{0}^{N}ds_{\alpha }\int_{0}^{N}ds_{\beta }U\left[R_{\alpha }\left(s_{\alpha }\right)-R_{\beta }\left(s_{\beta }\right)\right]\}$$

Here $F_{0}$ is the free energy of the background fluid where the polyzwitterions are present. It arises from the translational entropy of dissociated salt ions and solvent and the charge fluctuations from the salt ions, given by

(14) $$\frac{F_{0}}{k_{B}TV}=c_{0}\ln c_{0}-c_{0}+\sum_{i}\left(c_{i}\ln c_{i}-c_{i}\right)-\frac{\kappa^{3}}{12\pi },$$

where $c_{0}$ and $c_{i}$ are the number concentrations of the solvent and *i*-th electrolyte ion. In Equation (12),

(15) $$U\left(r\right)=v\ell^{3}\delta \left(r\right)+v_{dipole-dipole}\left(r,\kappa \right),$$

where *v* is the familiar excluded volume parameter, written equivalently as (1−2χ), where χ is the Flory-Huggins parameter to compound polymer-polymer, polymer-solvent, and solvent-solvent short-ranged interactions. The dipole contribution to U(r) is $v_{dipole-dipole}\left(r\right)$, as explicitly given in Equations (2)–(6). In the situation of anti-parallel orientations of two adjacent dipoles with a separation distance $r_{0}$ comparable to the monomer size *ℓ*, $v_{dipole-dipole}\left(r\right)$ can be written as a delta function (analogous to the treatment of the excluded volume interaction) given by

(16) $$v_{dipole-dipole}\left(r\right)=v_{d}\delta \left(r\right),$$

with

(17) $$v_{d}=-\frac{f\ell_{B}p^{2}}{r_{0}^{3}}e^{-\kappa r_{0}}\left(1+\kappa r_{0}\right),$$

where we introduce the probability *f* of realizing anti-parallel orientations of the dipoles of two adjacent zwitterions. Considering only the favored orientations (Figure 1c,d), *f* is 0.5, if the dipoles are completely free without any entropic contribution from chain connectivity. In realistic situations, we expect *f* to be smaller than this value. For example, based only on the loss of orientational entropy of a dipole to be oriented in a specified direction, f=1/4π. Combining Equations (15) and (17), we define an effective interaction parameter,

(18) $$v_{eff}=v+v_{d}=\left(1-2\chi \right)-\frac{f\ell_{B}p^{2}}{r_{0}^{3}}e^{-\kappa r_{0}}\left(1+\kappa r_{0}\right).$$

Hence, the χ parameter specific to polymer-solvent pair without zwitterions, is increased by an amount proportional to $\ell_{B}p^{2}$.

We note that the expression for $v_{eff}$ is quite different for the high temperature situation (upper part of Figure 1f) corresponding to randomly oriented dipoles. In this situation, $v_{eff}$ becomes [1]

(19) $$v_{eff}=v+v_{dd},$$

where

(20) $$v_{dd}=-\frac{\pi }{9}\frac{\ell_{B}^{2}p^{4}}{\ell^{6}}e^{-2\kappa \ell }\left[4+8\kappa \ell +4{\left(\kappa \ell \right)}^{2}+{\left(\kappa \ell \right)}^{3}\right].$$

Here the dipole contribution is proportional to $\ell_{B}^{2}p^{4}$, instead of $\ell_{B}p^{2}$ in Equation (17).

## 3. Size and Dynamics of Polyzwitterions in Dilute Solutions

### 3.1. Size: Anti-Polyelectrolyte Effect

For an isolated polyzwitterion chain, the probability distribution function G(R,N) for a chain of *N* repeat units and end-to-end distance R follows from Equations (9)–(20) as

(21) $$\begin{array}{c} G\left(R,N\right)=\int_{0}^{R}D\left[R\left(s\right)\right]\exp\{-\frac{3}{2\ell^{2}}\int_{0}^{N}ds{\left(\frac{\partial R(s)}{\partial s}\right)}^{2}-\frac{\ell^{3}}{2}\int_{0}^{N}ds\int_{0}^{N}ds^{\prime }v_{eff}\delta \left[R\left(s\right)-R\left(s^{\prime }\right)\right]\\ -\ell^{6}w\int_{0}^{N}ds\int_{0}^{N}ds^{\prime }\int_{0}^{N}ds^{\prime \prime }\delta \left[R\left(s\right)-R\left(s^{\prime }\right)\right]\delta \left[R\left(s^{\prime }\right)-R\left(s^{\prime \prime }\right)\right], \end{array}$$

where the three-body interaction of strength *w* is included in anticipation of the necessity to stabilize the expected globular state of the polyzwitterion chain. The free energy $F_{1}$ and the mean square end-to-end distance $\langle R^{2}\rangle$ of the chain are given by G(R,N) as

(22) $$\frac{F_{1}}{k_{B}T}=-\ln\int dR\;G\left(R,N\right),$$

and

(23) $$\langle R^{2}\rangle =\frac{\int dR\;R^{2}\;G\left(R,N\right)}{\int dR\;G(R,N)}.$$

We will use the variational procedure [44], which has been found to be effective in treating polyelectrolytes and neutral polymers, to calculate $F_{1}$ and $\langle R^{2}\rangle$ in terms of $\chi ,\ell_{B},p$, and $c_{s}$. Since the technical details of this procedure are already in the literature [1,44], we give only the results for $F_{1}$ and $\langle R^{2}\rangle$ along with the effect of dipoles on the theta θ temperature relevant to phase behavior.

#### 3.1.1. Designing θ Temperature

Writing $v_{eff}$ of Equation (18) as ($1-2\chi_{eff}$), we get

(24) $$1-2\chi_{eff}=1-2\chi -\frac{f\ell_{B}p^{2}}{r_{0}^{3}}e^{-\kappa r_{0}}\left(1+\kappa r_{0}\right),$$

so that

(25) $$\chi_{eff}=\chi +\frac{1}{2}\frac{f\ell_{B}p^{2}}{r_{0}^{3}}e^{-\kappa r_{0}}\left(1+\kappa r_{0}\right).$$

**Figure 2:**
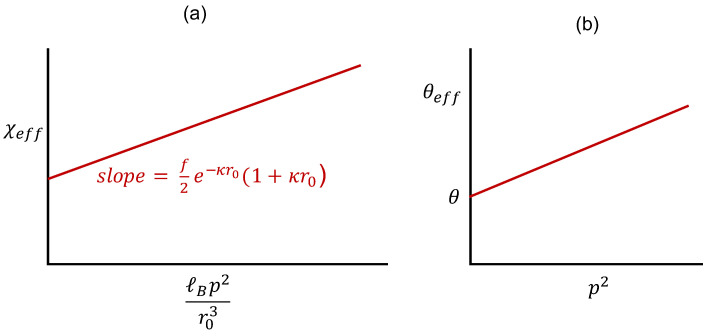
Elevation of θ temperature by $p^{2}$. (**a**) Effective chi parameter and (**b**) effective theta temperature.

Hence a plot of $\chi_{eff}$ versus $\ell_{B}p^{2}/\ell^{3}$ is linear with the slope depending on salt concentration as shown in Figure 2a. If the polyzwitterion solution exhibits upper solution critical solution temperature (UCST) behavior, as prescribed by χ=θ/2T, the dependence of θ temperature (now $\theta_{eff}$) on the dipole length of the zwitterion is given by

(26) $$\theta_{eff}=\theta +\frac{fe^{2}p^{2}e^{-\kappa r_{0}}\left(1+\kappa r_{0}\right)}{4\pi \epsilon_{0}\epsilon_{r}k_{B}r_{0}^{3}},$$

where the definition of $\ell_{B}$ is used. The elevation of the theta temperature is linearly proportional to $p^{2}$ with the coefficient being a decreasing function of $c_{s}$. Thus the dipole length *p* of the zwitterion has a significant effect on the θ temperature of polyzwitterion solutions and offers an important synthesis tool to design the phase behavior of polyzwitterions.

#### 3.1.2. Size and Free Energy

Using the variational procedure and following the derivation in Refs. [1,44], the free energy $F_{1}$ of a single polyzwitterion chain in solutions is obtained from Equation (22) as

(27) $$\frac{F_{1}}{k_{B}T}=\frac{3}{2}\left[\frac{R^{2}}{N\ell^{2}}-1-\ln\left(\frac{R^{2}}{N\ell^{2}}\right)\right]+\frac{4}{3}{\left(\frac{3}{2\pi }\right)}^{3/2}\left(v+v_{d}\right)\frac{N^{2}\ell^{3}}{R^{3}}+w\frac{N^{3}\ell^{6}}{R^{6}},$$

where the unknown three-body interaction parameter *w* absorbs all numerical prefactors. By minimizing the above free energy expression with respect to the root mean square end-to-end distance *R*, $dF_{1}\left(R\right)/dR=0$, we get

(28) $${\left(\frac{R^{2}}{N\ell^{2}}\right)}^{5/2}-{\left(\frac{R^{2}}{N\ell^{2}}\right)}^{3/2}=\frac{4}{3}{\left(\frac{3}{2\pi }\right)}^{3/2}\left(v+v_{d}\right)\sqrt{N}+2w{\left(\frac{N\ell^{2}}{R^{2}}\right)}^{3/2}.$$

Rewriting this result in terms of the chain expansion factor $\alpha ={\left(R^{2}/N\ell^{2}\right)}^{1/2}$, we obtain

(29) $$\alpha^{5}-\alpha^{3}-\frac{2w}{\alpha^{3}}=\frac{4}{3}{\left(\frac{3}{2\pi }\right)}^{3/2}\left(v+v_{d}\right)\sqrt{N}.$$

**Figure 3:**
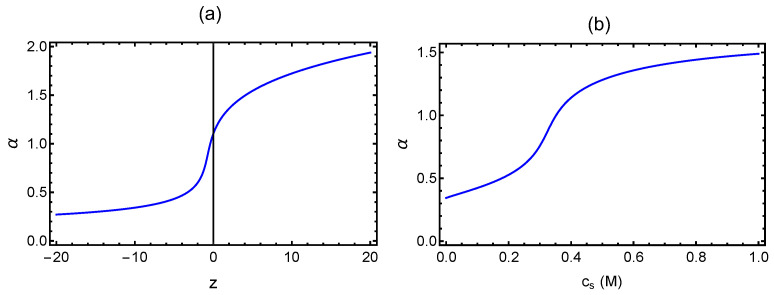
(**a**) Coil-globule transition: α is the chain expansion factor and $z=\frac{4}{3}{\left(\frac{3}{2\pi }\right)}^{3/2}\left(v+v_{d}\right)\sqrt{N}$. (**b**) Anti-polyelectrolyte effect.

A plot of α versus the term on the right-hand-side of this equation is given in Figure 3a exhibiting the coil-globule transition.

Since the dipole-dipole interactions are attractive ($v_{d}<0$), polyzwitterions can readily form globules for sufficiently large dipole lengths. In addition to the dipole length, $c_{s}$ plays an important role in determining the dipole-dipole interaction strength. Once a polyzwitterion forms a globule in the low salt condition, it expands upon weakening the inter-segment dipole-dipole attraction with additional salt. This behavior of formation of expanded coil from a globule upon addition of salt is illustrated in Figure 3b, where the chain expansion factor α is plotted versus $c_{s}$ for v=1,N=225,w=0.2, and $f\ell_{B}p^{2}/r_{0}^{3}=2.5$. This behavior is the opposite to the familiar polyelectrolyte behavior where the size shrinks due to the screening of charge-charge repulsion with added salt. The ‑anti-polyelectrolyte’ behavior of polyzwitterion chains arises from screening of the attractive dipole-dipole interaction with added salt. The extreme limits of formation of globule and coil structures and the intervening crossover behavior are given by Equation (29). The size and free energy of the coil and globule states predicted by Equations (27) and (28) are as follows.

(a) Polyzwitterion coils ($v+v_{d}>0$):

For sufficiently large positive values of $v+v_{d}$, Equation (28) gives

(30) $${\left(\frac{R^{2}}{N\ell^{2}}\right)}^{5/2}=\frac{4}{3}{\left(\frac{3}{2\pi }\right)}^{3/2}\left(v+v_{d}\right)\sqrt{N},$$

so that the root-mean-square end-to-end distance *R*, which is proportional to the radius of gyration $R_{g}$, scales with the degree of polymerization *N* as

(31) $$\frac{R_{g}}{\ell }∼{\left(v+v_{d}\right)}^{1/5}N^{3/5},$$

with the size exponent ν=3/5. This result is as expected for good solution conditions. Substituting this result in Equation (27), the free energy $F_{1}$ is given by

(32) $$\frac{F_{1}}{k_{B}T}=1.8{\left(v+v_{d}\right)}^{2/5}N^{1/5}.$$

(b) Polyzwitterion globules ($v+v_{d}<0$): For sufficiently strong attractive dipole-dipole interactions ($v+v_{d}<0$), Equation (28) gives

(33) $${\left(\frac{R^{2}}{N\ell^{2}}\right)}^{3/2}={\left[\frac{4}{3}{\left(\frac{3}{2\pi }\right)}^{3/2}\left|v+v_{d}\right|\right]}^{-1}\frac{2w}{\sqrt{N}},$$

so that

(34) $$\frac{R}{\ell }={\left[\frac{3}{2}{\left(\frac{2\pi }{3}\right)}^{3/2}\frac{w}{|v+v_{d}|}\right]}^{1/3}N^{1/3}.$$

Hence the scaling behavior of $R_{g}$ of polyzwitterion globule is

(35) $$\frac{R_{g}}{\ell }∼{\left(\frac{w}{|v+v_{d}|}\right)}^{1/3}N^{1/3},$$

with the size exponent ν=1/3, as expected for globules.

Substituting Equation (34) into Equation (27), the free energy of the globule is given as

(36) $$\frac{F_{1}}{k_{B}T}=\frac{9}{2\pi^{3}}\frac{|v+v_{d}{|}^{2}}{w}N.$$

### 3.2. Dynamics

The Rouse equation for the dynamics of the position $R_{m}\left(t\right)$ of the *m*-th segment at time *t*, applicable in the absence of hydrodynamic interaction, is given as [1,45]

(37) $$\zeta_{b}\frac{\partial R_{m}\left(t\right)}{\partial t}-\frac{3k_{B}T}{\ell^{2}}\frac{\partial^{2}R_{m}\left(t\right)}{\partial m^{2}}+\frac{\partial }{\partial R_{m}}\sum_{j}U\left(R_{m}-R_{j}\right)=f_{m}\left(t\right),$$

where $\zeta_{b}$ is the segment friction coefficient, *U* is the intersegment interactions (Equation (9)), and $f_{m}$ is the random force acting on the *m*-th segment. By approximately linearizing the nonlinear dependence of *U* on $R_{m}\left(t\right)$ in terms of an effective Kuhn length $\ell_{\mathrm{eff}}$, Equation (37) is approximated as [1]

(38) $$\zeta_{b}\frac{\partial R_{m}\left(t\right)}{\partial t}-\frac{3k_{B}T}{\ell \ell_{\mathrm{eff}}}\frac{\partial^{2}R_{m}\left(t\right)}{\partial m^{2}}=f_{m}\left(t\right),$$

where $\ell_{\mathrm{eff}}$ is related to the chain expansion factor α as $\ell_{\mathrm{eff}}/\ell =\alpha^{2}$ with $\langle R^{2}\rangle =N\ell \ell_{\mathrm{eff}}$. In general, since $\langle R^{2}\rangle ∼N^{2\nu }$,

(39) $$\ell_{\mathrm{eff}}∼N^{2\nu -1}.$$

Introducing the Fourier transform of $R_{m}\left(t\right)$ in terms of the Rouse modes ${\hat{R}}_{p}\left(t\right)$ (where *p* denotes the Rouse mode index),

(40) $$R\left(m,t\right)=\sum_{p=-\infty }^{\infty }{\hat{R}}_{p}\left(t\right)\cos\left(\frac{\pi pm}{N}\right),$$

(41) $${\hat{R}}_{p}\left(t\right)=\frac{1}{N}\int_{0}^{N}dm\;R\left(m,t\right)\cos\left(\frac{\pi pm}{N}\right),$$

we get

(42) $$\zeta_{b}\frac{\partial {\hat{R}}_{p}\left(t\right)}{\partial t}+\frac{3\pi^{2}k_{B}T}{\ell \ell_{\mathrm{eff}}}{\left(\frac{p}{N}\right)}^{2}{\hat{R}}_{p}\left(t\right)={\hat{f}}_{p}\left(t\right),\;\;\;\;\;\;\;\;\left(\mathrm{Rouse}\right)$$

where

(43) $$\ell_{\mathrm{eff}}∼{\left(\frac{\pi p}{N}\right)}^{1-2\nu }.$$

The dependence of the relaxation time $\tau_{p}$ of the *p*-th Rouse mode is obtained from Equation (42) as

(44) $$\tau_{p}=\frac{\zeta_{b}\ell \ell_{\mathrm{eff}}}{3\pi^{2}k_{B}T}{\left(\frac{N}{p}\right)}^{2}∼\frac{\zeta_{b}}{T}{\left(\frac{N}{p}\right)}^{2\nu +1}.$$

The longest chain relaxation time $\tau_{R}$ (Rouse time) corresponding to p=1 scales as

(45) $$\tau_{R}∼N^{2\nu +1}\;\;\;\;\;\;\;\;\;\;\left(\mathrm{Rouse}\right)$$

According to the Rouse dynamics, the time correlation function of the end-to-end distance vector $X\left(t\right)=R_{N}\left(t\right)-R_{0}\left(t\right)$ is known as [45]

(46) $$\langle X\left(t\right)\cdot X\left(0\right)\rangle =\langle R^{2}\rangle \left(\frac{8}{\pi^{2}}\right)e^{-t/\tau_{R}}∼e^{-A_{R}t/N^{2\nu +1}},\;\;\;\;\;\;\;\;\;\;\left(\mathrm{Rouse}\right)$$

where $A_{R}$ is the prefactor in Equation (44). The above equation is derived using the assumption that the longest relaxation time (p=1 mode) dominates the time correlation function of the end-to-end distance.

When hydrodynamic interaction is fully present as in dilute solutions, Equation (42) is modified into the Zimm description [1],

(47) $$\frac{\partial {\hat{R}}_{p}\left(t\right)}{\partial t}+D_{p}\frac{3\pi^{2}k_{B}T}{\ell \ell_{\mathrm{eff}}}{\left(\frac{p}{N}\right)}^{2}{\hat{R}}_{p}\left(t\right)=\frac{{\hat{f}}_{p}\left(t\right)}{\zeta_{b}}.\;\;\;\;\;\;\;\;\left(\mathrm{Zimm}\right)$$

Here, $D_{p}$ is the preaveraged hydrodynamic interaction tensor for all chain segments given as

(48) $$D_{p}∼\frac{1}{\eta_{0}}{\left(\frac{\pi p}{N}\right)}^{\nu -1}.$$

The Zimm relaxation time of the *p*-th Rouse mode follows from Equation (47) as

(49) $$\tau_{p,\mathrm{Zimm}}=\frac{\ell \ell_{\mathrm{eff}}}{3\pi^{2}k_{B}T}\frac{1}{D_{p}}{\left(\frac{N}{p}\right)}^{2}.$$

Using Equations (43) and (48),

(50) $$\tau_{p,\mathrm{Zimm}}∼\frac{\eta_{0}}{T}{\left(\frac{N}{p}\right)}^{3\nu },$$

so that the longest relaxation time $\tau_{Z}$ (Zimm time) is

(51) $$\tau_{Z}∼N^{3\nu }.\;\;\;\;\;\;\;\;\;\;\left(\mathrm{Zimm}\right)$$

Analogous to Equation (46), the time correlation function of the end-to-end distance vector X(t) with hydrodynamic interaction present is given by

(52) $$\langle X\left(t\right)\cdot X\left(0\right)\rangle ∼e^{-t/\tau_{Z}}∼e^{-A_{Z}t/N^{3\nu }}.\;\;\;\;\;\;\;\;\;\;\left(\mathrm{Zimm}\right)$$

The above results on polyzwitterion single chains are used below in arriving at mean field predictions of microgel formation and internal dynamics of gels in polyzwitterion systems.

## 4. Polyzwitterion Microgels in Solutions

For polyzwitterion concentrations above the overlap concentration, the chains interpenetrate and intermingle. For such semidilute concentrations, the solution is uniform if there are no driving forces for local aggregation. in the case of polyzwitterions, such a scenario is feasible only in the high temperature phase in Figure 1f where the zwitterion dipoles are randomly oriented. On the other hand, in the low temperature phase (bottom in Figure 1e), the stable quenched quadrupoles are expected to result in pervasive network formation with local heterogeneous structures and macrophase separation. At intermediate conditions for $T\geq T_{threshold}$, we anticipate formation of physical aggregation into microgels.

We label this situation as the mesomorphic state since it emerges as an interlude between homogeneous liquid phase and liquid-liquid or liquid-gel macrophase separation. In the mesomorphic state of polyzwitterion solutions, most of the zwitterion dipoles are randomly oriented with the rest of them are anchored into quadrupoles. The quadrupole energy can be quite high compared to the thermal energy. As an example, for ℓ=1nm, p=1nm, $\ell_{B}=0.7\;\mathrm{nm}$, and the inter-dipole distance $r_{0}=0.25\;\mathrm{nm}$, the quadrupole energy *u* (Figure 1e) is $64k_{B}T$. If *u* is so strong, the various quadrupoles will act like physical cross-links and the intervening strands behave like flexible chains with intra-strand excluded volume interactions and randomly oriented dipole-dipole interactions.

We address this mesomorphic state by the following approach. (a) Using combinatorics and the pioneering works of Tanaka [46] and Semenov and Rubinstein [47] on associating polymers, we present the equation of state for the fraction of dipoles involved in quadrupole formation. (b) Using Flory’s mean field theory [48,49,50,51,52] of swollen networks, swelling equilibrium of polyzwitterion gels is derived. (c) Specializing on microgels formed by finite number of polyzwitterion chains, their free energy is derived. (d) Using the free energies of isolated chains and microgels containing prescribed number of chains, an the classical micellization/aggregation theory [53], the critical aggregation concentration (CAC) and microgel size distribution are derived.

### 4.1. Equation of State for Quadrupole Formation

For a solution of volume $V_{0}$ containing *n* polyzwitterion chains, each with *N* repeat units of volume $v_{0}$, the total number of dipoles capable of quadrupole formation is

(53) $$N_{s}=fnN=f\phi_{0}\frac{V_{0}}{v_{0}},$$

where *f* is the fraction of monomers that have the correct orientation for the formation of quadrupoles, and $\phi_{0}=nNv_{0}/V_{0}$ is the volume fraction of polyzwitterion. Let *q* be the fraction of dipoles associated as quadrupoles so that the number of physical crosslinks $N_{c}$ is given as

(54) $$N_{c}=\frac{q}{2}fnN=\frac{q}{2}N_{s}.$$

The free energy $F_{0}$ of the cross-linked Gaussian network is

(55) $$\frac{F_{0}}{k_{B}T}=-\ln Z_{0},$$

where the partition function $Z_{0}$ is given by

(56) $$Z_{0}=Q_{1}Q_{2}Q_{3}e^{\epsilon N_{c}},$$

where $Q_{1},Q_{2},$ and $Q_{3}$ are, respectively, the probability to choose $2N_{c}$ dipoles out of $N_{s}$ dipoles, the probability of forming $N_{c}$ quadrupoles out of $2N_{c}$ dipoles, and the probability of finding two dipoles closeby. ϵ is the energy gain in the formation of one quadrupole. $Q_{1},Q_{2},$ and $Q_{3}$ are given by

(57) $$Q_{1}=\frac{N_{s}!}{\left(2N_{c}\right)!\left(N_{s}-2N_{c}\right)!},\;\;Q_{2}=\left(2N_{c}-1\right)\left(2N_{c}-3\right)\cdots 3\cdot 1,Q_{3}={\left(\frac{v_{0}}{V_{0}}\right)}^{N_{c}}.$$

Substituting Equations (56) and (57) in Equation (55), and using the Stirling approximation, we get

(58) $$\frac{F_{0}}{k_{B}T}=-\left(\frac{v_{0}}{V_{0}}\right)\left[\frac{fq\phi_{0}}{2}\ln\left(\frac{f\phi_{0}}{e}\right)-\frac{f\phi_{0}}{2}\left[q\ln q+2\left(1-q\right)\ln\left(1-q\right)-\epsilon q\right]\right].$$

Minimizing $F_{0}$ with respect to *q*, $\partial F_{0}/\partial q=0$, we get the equation of state for the optimal fraction of dipoles associated into quadrupoles as

(59) $$\frac{q}{{\left(1-q\right)}^{2}}=f\phi_{0}e^{\epsilon }.$$

This expression is equivalent to the previous results of Semenov and Rubinstein [47], and the chemical equilibrium expression of Tanaka [46].

### 4.2. Swelling Equilibrium of Polyzwitterion Gels

We assume that the free energy *F* of a gel made from *n* chains (of *N* monomers) is the additive sum of free energy $F_{el}$ due to elasticity, free energy of mixing $F_{mix}$, free energy $F_{elec}$ due to electrostatic interactions among randomly oriented dipoles, and free energy $F_{quad}$ due to formation of quadrupoles,

(60) $$F=F_{el}+F_{mix}+F_{elec}+F_{quad}.$$

The free energy $F_{el}$ from the elasticity of the gel is obtained from the earlier works of Flory, Dusek and Patterson, and Tanaka [48,49,50,51,52]. Given that there are $2N_{c}$ monomers involved in quadrupoles (crosslinks) out of the total $N_{0}=nN$ monomers in the system, the number of monomers per cross-linked unit, $N_{e}$, is

(61) $$N_{e}=\frac{N_{0}}{2N_{c}}=\frac{nN}{2N_{c}}.$$

Adopting the Flory-Stockmayer assumption that the cross-linking statistics is that of tree-like network architecture, the number of elastically effective chain strand ($n_{eff}$) is

(62) $$n_{eff}=2N_{c}\left(1-\frac{2N_{c}}{N}\right).$$

Approximating this result,

(63) $$n_{eff}≃2N_{c}=\frac{nN}{N_{e}}=\frac{n}{\gamma },$$

where Equation (61) is used and $\gamma =N_{e}/N$. Thus there are $n_{eff}$ elastically active strands of $N_{e}$ monomers and $N_{c}=n_{eff}/2$ crosslinks.

It is noteworthy to point out that the minimum value of *N*, $N_{min}$, required for network formation depends inversely on the fraction *q* of dipoles associated as quadrupoles. Since $N_{c}=qfnN/2$ (Equation (54)),

(64) $$N_{e}=\frac{nN}{2N_{c}}=\frac{1}{qf},$$

so that $N_{min}=N_{e}$ given as

(65) $$N_{min}==\frac{1}{qf},$$

where the equilibrium value of *q* is given in Equation (59).

For an isotropically swollen gel of swelling ratio $\lambda =V/V_{0}$ (where *V* is the volume of the swollen gel and $V_{0}$ is the gel volume in its reference state of Gaussian network), the standard theory of rubber elasticity gives

(66) $$\frac{F_{el}}{k_{B}T}=\frac{3}{2}n_{eff}\left(\lambda^{2}-1-\ln\lambda \right).$$

The volume fraction of the polyzwitterion in the swollen gel is

(67) $$\phi =\frac{V_{d}}{V}=\frac{V_{d}}{V_{0}}=\phi_{0}\frac{1}{\lambda^{3}},$$

where $V_{d}$ is the volume of dry polyzwitterion material and $\phi_{0}=V_{d}/V_{0}$. Taking $V_{d}=nNv_{0}$ and $V_{0}$ as the value given by the volume of Gaussian coils proportional to $N^{3/2}$,

(68) $$\phi_{0}=\frac{nNv_{0}}{n(4\pi R_{g0}^{3}/3)}=\frac{a}{\sqrt{N}},$$

where *a* is a constant of order one. In view of $\gamma =N_{e}/N$,

(69) $$\phi_{0}=a\sqrt{\frac{\gamma }{N_{e}}}.$$

By combining Equations (66) and (67), $F_{el}$ is given by

(70) $$\frac{F_{el}}{k_{B}T}=\frac{3}{2}n_{eff}\left[{\left(\frac{\phi_{0}}{\phi }\right)}^{2/3}-1+\frac{1}{3}\ln\left(\frac{\phi_{0}}{\phi }\right)\right].$$

For ϕ≪1, this expression reduces to

(71) $$\frac{F_{el}}{k_{B}T}=\frac{3}{2}n_{eff}{\left(\frac{\phi_{0}}{\phi }\right)}^{2/3}.$$

The classical expression for for the free energy of mixing for a gel is

(72) $$\frac{F_{mix}}{k_{B}T}=\left(\frac{V}{v_{0}}\right)\left[(1-\phi )\ln(1-\phi )+\chi \phi (1-\phi )\right],$$

where ϕ refers to $n_{eff}N_{e}v_{0}/V$.

The electrostatic contribution from randomly oriented zwitterion dipoles to the free energy is

$$\frac{F_{elec}}{k_{B}T}=\frac{\ell^{3}}{2}\sum_{\alpha }^{n_{eff}}\sum_{\beta }^{n_{eff}}\int_{0}^{N_{e}}ds_{\alpha }\int_{0}^{N_{e}}ds_{\beta }\;v_{dd}\;\delta \left[R\left(s_{\alpha }\right)-R\left(s_{\beta }\right)\right],$$

where mean field approximation is used and $v_{dd}$ is given in Equation (20). Combining $F_{mix}$ and $F_{elec}$, we get

(73) $$\frac{F_{mix}}{k_{B}T}+\frac{F_{elec}}{k_{B}T}=\frac{V}{v_{0}}\left[\left(1-\phi \right)\ln\left(1-\phi \right)+\chi \phi -\chi_{eff}\phi^{2}\right],$$

where $\chi_{eff}$ now is

(74) $$\chi_{eff}=\chi -v_{dd}/2.$$

For ϕ≪1,

(75) $$\frac{F_{mix}}{k_{B}T}+\frac{F_{elec}}{k_{B}T}=n_{eff}N_{e}\left[\left(\frac{1}{2}-\chi_{eff}\right)\phi +O\left(\phi^{2}\right)\right]+\mathrm{constant}.$$

The free energy due to quadrupole formation is

(76) $$\frac{F_{quad}}{k_{B}T}=-N_{c}\epsilon =-\frac{n_{eff}}{2}\epsilon$$

Collecting the results for $F_{el},F_{mix},F_{elec}$, and $F_{quad}$ from Equations (71), (75) and (76), we get the free energy of a gel with $n_{eff}$ elastically active strands as

(77) $$\frac{F_{n_{eff}}}{k_{B}Tn_{eff}}=-\frac{\epsilon }{2}+N_{e}\left(\frac{1}{2}-\chi_{eff}\right)\phi +\frac{3}{2}{\left(\frac{\phi_{0}}{\phi }\right)}^{2/3}.$$

The minimization of this expression with respect to ϕ yields the polyzwitterion volume fraction at the swelling equilibrium of the polyzwitterion gel as

(78) $$\phi =\frac{\phi_{0}^{2/5}}{N_{e}^{3/5}{\left(\frac{1}{2}-\chi_{eff}\right)}^{3/5}}.$$

Using Equation (68), we get

(79) $$\phi =\frac{a^{2/5}\gamma^{1/5}}{N_{e}^{4/5}{\left(\frac{1}{2}-\chi_{eff}\right)}^{3/5}}.$$

Substituting this result in Equation (77), we obtain

(80) $$\frac{F_{n_{eff}}}{k_{B}Tn_{eff}}=-\frac{\epsilon }{2}+\frac{5}{2}a^{2/5}\gamma^{1/5}{\left(\frac{1}{2}-\chi +\frac{v_{dd}}{2}\right)}^{2/5}N_{e}^{1/5}+\mathrm{constant}.$$

### 4.3. Equilibrium Distribution of Microgel Size

Since we know expressions for the free energies of isolated chains and microgels containing a certain number of polyzwitterion chains, albeit using mean field arguments, we can derive the distribution of number of polyzwitterion chains in aggregates as a function of the characteristics of the zwitterion dipoles, and salt concentration for $T\geq T_{threshold}$. According to the standard theory [53] of micellization/aggregation, the mole fraction $X_{m}$ of aggregates with *m* elastically effective strands ($mN_{e}=nN$) is given as

(81) $$X_{m}=m{\left[X_{1}e^{\left(F_{1}-\frac{F_{m}}{m}\right)/k_{B}T)}\right]}^{m},$$

with the constraint of conservation of the total mole fraction *X* as

(82) $$X=\sum_{m=1}^{\infty }X_{m}.$$

In Equation (81), $X_{1}$ is the mole fraction of unaggregated polyzwitterion chains and $F_{1}$ is given by Equation (32) by replacing $v_{d}$ by $v_{dd}$. $F_{m}$ is given by Equation (80) by replacing $n_{eff}$ by *m*. In the context of aggregation equilibrium depicted in Equation (81), the constant term in Equation (80) is not arbitrary. We choose this constant term such that $F_{m}\to F_{1}$ for m=1 in order to comply with the constraint in Equation (82), $X_{m=1}=X_{1}$. This yields

(83) $$\frac{1}{k_{B}T}\left(F_{1}-\frac{F_{m}}{m}\right)=\left(1-\frac{1}{m}\right)\Theta ,$$

where Θ follows from Equation (32) (with $v_{d}$ replaced by $v_{dd}$), and Equation (80), as

(84) $$\Theta =\frac{\epsilon }{2}+\left(2.375-\frac{5}{2}a^{2/5}\gamma^{1/5}\right){\left(\frac{1}{2}-\chi +\frac{v_{dd}}{2}\right)}^{2/5}N_{e}^{1/5}.$$

Substitution of Equation (83) in Equation (81) gives

(85) $$X_{m}=m{\left(X_{1}e^{\Theta }\right)}^{m}\;e^{-\Theta }.$$

This indicates that the approximate value of the critical aggregation concentration (CAC) is

(86) $$\mathrm{CAC}=e^{-\Theta }.$$

The convenient form of $X_{m}$ given by Equation (85) enables analytical derivation of $X_{1}$, the average number of chains and the number of chains with the maximum propensity in the microgels. Substituting Equation (85) in Equation (82), we get

(87) $$X=\sum_{m=1}^{\infty }X_{m}=\sum_{m=1}^{\infty }m{\left(X_{1}e^{\Theta }\right)}^{m}e^{-\Theta }=\frac{X}{{\left(1-X_{1}e^{\Theta }\right)}^{2}}.$$

Solving the quadratic equation for $X_{1}$ in terms of *X* and Θ in Equation (87), we obtain

(88) $$X_{1}=\frac{\left(1+2Xe^{\Theta }\right)-\sqrt{1+4Xe^{\Theta }}}{2Xe^{2\Theta }}.$$

### 4.4. Representative Results

**Figure 4:**
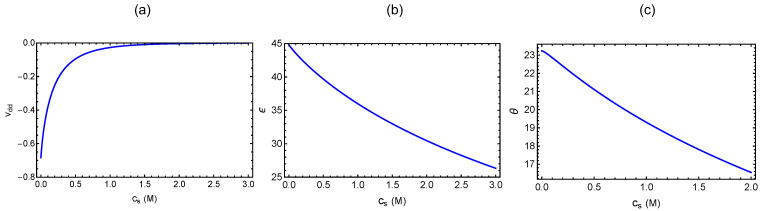
Dependence on salt concentration $c_{s}$ of (**a**) $v_{dd}$, (**b**) ϵ, and (**c**) Θ.

For the purpose of illustrating the general features of the dependence of $X_{m}$ on the number of chains in the microgel on the polyzwitterion concentration *c* and monovalent salt concentration $c_{s}$, we give the following results. In Equation (84), Θ depends on $v_{dd}$ and ϵ, which are $c_{s}$ dependent, and *N* ($=N_{e}/\gamma$). By choosing $a=1,\gamma =0.1,N_{e}=50$, χ=0, $\ell_{B}=0.7\;\mathrm{nm},p=1\;\mathrm{nm},\ell =1\;\mathrm{nm}$, and $\kappa \ell =2.26\sqrt{c_{s}}$, $v_{dd}$ is plotted in Figure 4a as a function of $c_{s}$ in molarity. For the antiparallel quadrupole, we choose $\ell_{B}=0.7\;\mathrm{nm},p=1\;\mathrm{nm}$, and $r_{0}=0.25\;\mathrm{nm}$, and ϵ, given by $\left(\ell_{B}p^{2}/r_{0}^{3}\right)\left(1+\kappa r_{0}\right)\exp\left(-\kappa r_{0}\right)$, is given in Figure 4b as a function of $c_{s}$. For the above choice of parameter values, the $c_{s}$ dependence of Θ is given in Figure 4c.

**Figure 5:**
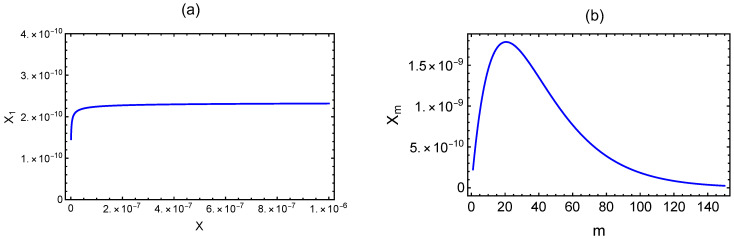
(**a**) Dependence of the mole fraction $X_{1}$ of unaggregated polyzwitterions on the total mole fraction of the polyzwitterion chains. (**b**) Distribution of the mole fraction of microgels containing *m* chains on *m* for c= 1 g/L and $c_{s}=0.25$ M.

**Figure 6:**
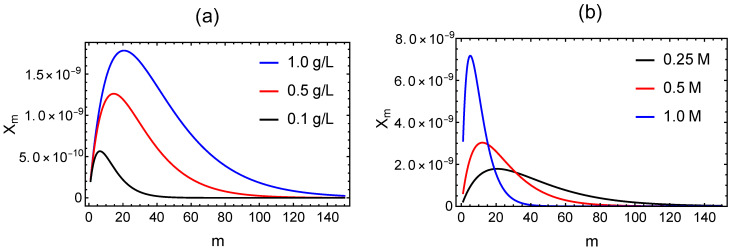
(**a**) Polyzwitterion concentration dependence of the distribution of the mole fraction of microgels containing *m* chains on *m* for c= 1.0, 0.5, and 0.1 g/L and $c_{s}=0.25$ M. (**b**) Salt concentration dependence of the distribution of the mole fraction of microgels containing *m* chains on *m* for $c_{s}=$ 0.25, 0.5, and 1.0 M and c= 1.0 g/L.

For $c_{s}=0.25$ M, the dependence of the mole fraction of unaggregated chains $X_{1}$ on the total mole fraction *X* of polyzwitterion chains is given in Figure 5a. The occurrence of CAC ($e^{-\Theta }$) is evident from this figure. Considering poly(sulfobetaine methacrylate) as a typical polyzwitterion, with N=512 and $M_{w}=180,216$ g/mol, the mole fraction of the polymer is related to its concentration *c* in g/L as $X=10^{-7}=c$. The distribution of $X_{m}$ on *m* for $c_{s}=0.25$ M and c=1 g/L is displayed in Figure 5b exhibiting the spontaneous selection of finite-sized microgels with a maximum around m=20. The dependence of $X_{m}$ on *c* (1.0, 0.5, 0.1 g/L) at the fixed salt concentration $c_{s}=$ 0.25 M is portrayed in Figure 6a. At lower polyzwitterion concentrations, the propensity of formation of microgels is lower with the corresponding microgel decreasing continuously. The dependence of $X_{m}$ on $c_{s}$ is illustrated in Figure 6b at c=1 g/L for $c_{s}=$ 0.25 M, 0.5 M, and 1.0 M. The microgels are constituted by smaller number of chains as the salt concentration increases. The above quantitative predictions on the self-assembly of polyzwitterion microgels can be tailored to various polyzwitterion systems by choosing appropriate values of the system-dependent parameters such as the dipole moment.

Using Equation (85) for $X_{m}$ at X> CAC, $X_{m}$ simplifies to

(89) $$X_{m}≃m\exp\left(-\frac{m}{\sqrt{Xe^{\Theta }}}-\Theta \right),$$

so that $\partial X_{m}/\partial m=0$ yields the number of chains in the microgel with maximum probability as

(90) $$m_{max}=\sqrt{X}e^{\Theta /2},$$

and the average number of chains in the microgel as

(91) $$\langle m\rangle =2m_{max}=2\sqrt{X}e^{\Theta /2}.$$

Furthermore, taking the polyzwitterion volume fraction in the aggregate (microgel) as

(92) $$\phi_{agg}=\frac{\langle m\rangle N_{e}\ell^{3}}{\frac{4}{3}\pi R_{g,agg}^{3}},$$

where $R_{g,agg}$ is the average radius of gyration of the aggregate. Substituting $\phi_{agg}$ from Equation (79) in the above equation, we get

(93) $$R_{g,agg}={\left(\frac{3}{2\pi }\right)}^{1/3}e^{\Theta /6}{\left(\frac{1}{2}-\chi_{eff}\right)}^{1/5}N_{e}^{3/5}\ell X^{1/6}.$$

Hence the radius of gyration of the aggregate is proportional to the one-sixth power of polyelectrolyte concentration at all salt levels of self-assembly,

(94) $$R_{g,agg}∼c^{1/6},$$

with the prefactor proportional to the radius of gyration of a single isolated strand under the same experimental conditions of microgel formation. The above theoretically derived exponent 1/6 for polyzwitterion microgels has earlier been implicated in the contexts of the slow mode in salt-free polyelectrolyte solutions [40] and physical polyzwitterions [30] arising from counterion binding to polyelectrolytes. These references provide experimental validation of the predictions from the present theory. The above scaling behavior appears to be universal for all associative charged macromolecular systems.

## 5. Swelling Equilibrium and Internal Dynamics of Polyzwitterion Gels

When polyzwitterion molecules in a solution at concentrations above the overlap concentration are allowed to form permanent chemical crosslinks, such gels can exhibit rich behavior encompassing both the high-temperature and low-temperature attributes described in Introduction.

### 5.1. Swelling Equilibrium

The derivation given in Section 4 is valid for the present situation as well, where $N_{c}$ is the number of permanent crosslinks instead of physical quadrupoles (which might contribute to additional physical crosslinks). Therefore, the swelling equilibrium is given by Equation (78) as

(95) $$\phi =\frac{\phi_{0}^{2/5}}{N_{e}^{3/5}{\left(\frac{1}{2}-\chi_{eff}\right)}^{3/5}},$$

where

(96) $$\chi_{eff}=\chi -\frac{1}{2}v_{dd},$$

for the experimental conditions that allow random orientations of the zwitterion groups, and

(97) $$\chi_{eff}=\chi -\frac{1}{2}v_{d},$$

for conditions where quenched quadrupoles are preferred. Since the quantitative consequences of $v_{d}∼p^{2}$ and $v_{dd}∼p^{4}$ can be significant, the volume phase transitions of chemically cross-linked polyzwitterion gels and their response to externally imposed stimuli such electric fields, mechanical forces, and added salt, can be quite different for the above two scenarios. While these issues are not addressed in the present paper, such a premise is of interest for future considerations.

### 5.2. Internal Dynamics

The displacement vector of the gel is a fluctuating quantity, shich can be quantified, for example, by a combination dynamic light scattering (DLS) experiments and gel elasticity theory. The displacement vector is directly proportional to to the end-to-end distance vector $X(N_{e},t)$ of the elastically active strands of $N_{e}$ monomers in the gel. As already shown above in Equations (46) and (52), the time correlation function of $X(N_{e},t)$ is

(98) $$\langle X\left(t\right)\cdot X\left(0\right)\rangle ∼\exp\left(-\frac{t}{\tau_{0}N_{e}^{x}}\right),$$

where $\tau_{0}$ absorbs all numerical prefactors and

(99) $$x=\left\{\begin{array}{cc} 2\nu +1 & \mathrm{Rouse}\\ 3\nu & \mathrm{Zimm} \end{array}\right.$$

In almost all chemically cross-linked gels, $N_{e}$ of all elastically active strands is not the same. For weakly cross-linked gels, it is well known that $N_{e}$ is distributed according to the exponential [54],

(100) $$P\left(N_{e}\right)=e^{-kN_{e}},$$

where *k* is the rate of the cross-linking reaction. In fact, even if the segments between two adjacent cross-links were to locally organize into vitrimer-like dynamically associated structures, the form of Equation (100) is preserved with *k* depending on the local dynamical details. The electric field (E) correlation function $g_{1}\left(t\right)$ measured in DLS is the superposition of Equations (98) and (100),

(101) $$g_{1}\left(t\right)∼\int dN_{e}\;e^{-kN_{e}-\frac{t}{\tau_{0}N_{e}^{x}}}∼e^{-{\left(\frac{t}{\tau }\right)}^{\beta }},$$

where τ is the net characteristic time that compounds all hierarchical relaxation times for strands with varying $N_{e}$ values, and β is a stretched exponent,

(102) $$\beta =\frac{1}{1+x}.$$

If hydrodynamic interactions are absent, then the Rouse dynamics is applicable and we get from Equation (99),

(103) $$\beta =\frac{1}{2\nu +2}.$$

On the other hand, if the gel meshes are widely open to allow hydrodynamic interaction without significant screening, the Zimm dynamics is applicable so that

(104) $$\beta =\frac{1}{3\nu +1}.$$

**Figure 7:**
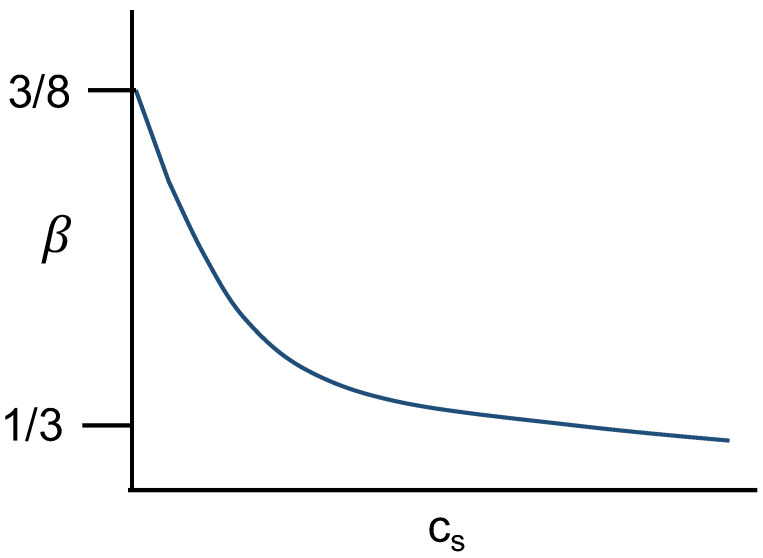
The stretched exponent β for hierarchical internal dynamics of polyzwitterion gels is 8/3 for salt-free limit and it decreases to 1/3 as the concentration of added salt increases.

For polyzwitterion gels, where the local polymer concentration is rather high, we expect that the hydrodynamic interaction is screened so that the Rouse dynamics is applicable. As already described, when the quadrupole formation is prevalent at low salt concnetrations, ν=1/3 so that β=8/3. On the other hand, if the formation of quadrupoles is weakened by adding salt, and since the strands between crosslinks are not too long to allow significant excluded volumere swelling, we expect ν≃1/2 so that β=1/3. Thus we anticipate a crossover from β=8/3 to β=1/3 as $c_{s}$ is increased for the hierarchical internal dynamics of polyzwitterion gels as sketched in Figure 7.

## 6. Conclusions

The incipient ability of dipolar zwitterion monomers of polyzwitterions to associate results in a broad range of properties unlike the situation with uniformly charged polyelectrolytes. Even though there have previously been several theoretical attempts to treat polyzwitterions, they assume that the dipolar zwitterionic moieties are randomly oriented. In reality, this assumption can be valid only if the temperature is extremely high or the concentration of added low molar mass electrolyte is very high. In order to be relevant to practical experimental systems, it is necessary to treat drastic deviations from the random-orientation paradigm. The theory presented here is a new paradigm for treating the behavior of polyzwitterions. Here, the non-random orientations of zwitterionic groups into quenched conformations are explicitly treated using statistical mechanics and field-theoretic methods.

In the present theory, two regimes have been identified for the rotational degree of freedom associated with the zwitterion dipoles. In the high-temperature regime, the dipoles are randomly oriented. In the low-temperature regime, the dipole orientations are quenched resulting in quadrupoles. In developing a theoretical formulation for polyzwitterion solutions, we have accounted for the spontaneous formation of quadrupoles in the low-temperature regime.

In the present mean field theory of polyzwitterion systems, we have derived closed-form formulas for (a) the size and dynamics of isolated chains in dilute solutions, (b) self-assembly of mesomorphic microgels in semidilute solutions, and (c) swelling equilibrium and hierarchical internal dynamics of chemically cross-linked polyzwitterion gels.

**Figure 8:**
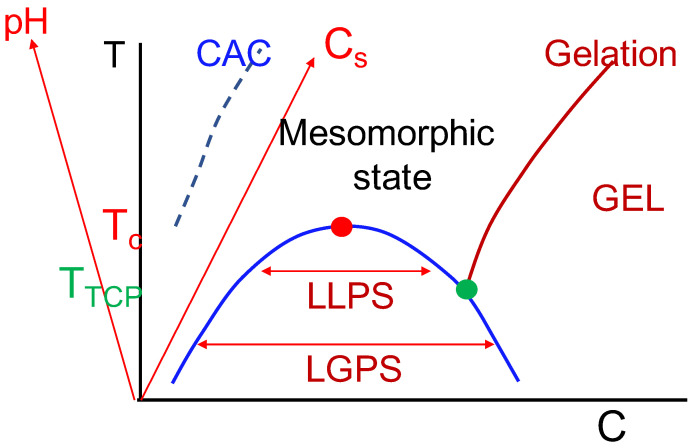
Generalized phase diagram for polyzwitterion solutions. The ordinate is any one of the variables: temperature *T*, salt concentration $c_{s}$, and pH of the solution. In general, the phase diagram is multi-dimensional. In order to emphasize this aspect of multi-dimensionality, we have inserted the slanted lines denoting pH and $c_{s}$ by focussing on the variable *T* (orthogonal to the abscissa denoting the polymer concentration).

The general picture that emerges from the considerations in the present work is sketched in Figure 8. The key experimental variables can be temperature or salt concentration or pH, in addition to the polyzwitterion concentration. In this figure, the ordinate is any one of the variables: temperature *T*, salt concentration $c_{s}$, and pH of the solution. In general, the phase diagram is multi-dimensional. In order to emphasize this aspect of multi-dimensionality, we have inserted the slanted lines denoting pH and $c_{s}$ by focussing on the variable *T* (orthogonal to the abscissa). Therefore, as an example of the ordinate variable, let the temperature be the experimental handle to explore the behavior of polyzwitterions. In extremely dilute solutions and at high temperatures, individual polyzwitterion chains exist as expanded coils. When the temperature is lowered, the chain undergoes coil-to-globule transition. However, the globule exhibits the anti-polyelectrolyte effect of expanding into the coil conformation upon addition of salt.

In the high-temperature region, but not too high, as the polymer concentration is increased, the polyzwitterion chains spontaneously aggregate into mesomorphic microgels. In between the dilute regime and the mesomorphic state, there is a line denoting the critical aggregation concentration necessary for the formation of the microgels. Upon further increase in polymer concentration, the mesmorphic state becomes a gel if the polymer concentration is higher than that corresponding to the gelation threshold.

For intermediate polymer concentrations, when the temperature is lowered, dipolar correlations result in quadrupoles and multipoles generating structural heterogeneity that interferes with fluctuations in the local polymer concentration. Eventually, as the temperature is lowered, a critical temperature $T_{c}$ is reached below which liquid-liquid phase separation (LLPS) occurs. At temperatures lower than $T_{c}$, a tricritical point can emerge at the temperature $T_{\mathrm{TCT}}$ at which the gel line meets the coexistence curve. For temperatures below $T_{\mathrm{TCT}}$, liquid-gel phase separation (LGPS) occurs.

In the above general context of the rich phase behavior, we have only addressed in this paper the behaviors outside the critical point and the coexistence curve. Since the present work shows that the dipolar associations in polyzwitterion systems lead to significantly rich consequences in contrast with considerations where dipoles are assumed to be randomly oriented, such dipolar associations are expected to play a major role in the construction of LLPS and LGPS phase behaviors.

This theory makes many predictions on (a) the structure and dynamics of polyzwitterions in dilute solutions, (b) emergence of a spontaneous formation of mesomorphic microgel aggregates and their size distribution, and (c) swelling equilibria and hierarchical internal dynamics of polyzwitterion gels. These predictions open a new avenue of experiments toward critical assessments of theoretical predictions, more fundamental understanding of polyzwitterion behavior, and potential applications.in health care industry. Furthermore, since the present theory is only based on mean-field arguments, treatment of concentration fluctuations is of immediate future interest.

It must be mentioned that the dipolar behaviors addressed in the present work are also present in biological macromolecules such as intrinsically disordered proteins. Extension of the present theory to such broader situations is of considerable future interest.

## Data Availability

The data presented in this study are openly available in article.
